# Hydrothermal Synthesis of Nanostructured Vanadium Oxides

**DOI:** 10.3390/ma3084175

**Published:** 2010-08-02

**Authors:** Jacques Livage

**Affiliations:** Chimie de la Matière Condensée, Collège de France, 11 place Marcelin Berthelot, 75231 Paris, France; E-Mail: jacques.livage@upmc.fr; Tel.: 33 (0)1 44 27 15 00; Fax: 33 (0)1 44 27 15 04

**Keywords:** vanadium oxide, nanostructure, aqueous chemistry

## Abstract

A wide range of vanadium oxides have been obtained via the hydrothermal treatment of aqueous V(V) solutions. They exhibit a large variety of nanostructures ranging from molecular clusters to 1D and 2D layered compounds. Nanotubes are obtained via a self-rolling process while amazing morphologies such as nano-spheres, nano-flowers and even nano-urchins are formed via the self-assembling of nano-particles. This paper provides some correlation between the molecular structure of precursors in the solution and the nanostructure of the solid phases obtained by hydrothermal treatment.

## 1. Introduction

The properties of solid state materials mainly depend on their structure and morphology. Therefore nanostructured materials are becoming very popular. They offer a great potential for improving properties and find applications in many fields such as microelectronics, batteries, sensing devices, nanoprobes and even nanomedicine [1,2,3,4]. Many nanostructured materials have been described during the past few years. Among them nanostructured vanadium oxides have been extensively studied since the discovery of VOx nanotubes by R. Nesper and his group [5,6]. They exhibit a great variety of nanostructures, ranging from 1D to 3D [7] and V_2_O_5_ has even been chosen as a model system for the description of nanostructured materials. Vanadium oxides find major applications in the field of lithium ion batteries [8,9,10,11].

One of the main points for a real development of nanostructured materials would be a better understanding of their formation in order to be able to build taylor-made nanostructures. The usual solid state synthesis of vanadium pentoxide via the thermal decomposition of ammonium metavanadate leads to the well known orthorhombic oxide α-V_2_O_5_ that exhibits a layered structure made of edge and corner sharing [VO_5_] double chains [12]. Many other structures based on vanadium oxide have been described in the literature [13,14]. Different types of vanadium coordination polyhedra such as trigonal bipyramids to square pyramids and tetrahedra are observed. Moreover, V^5+^ can be easily reduced leading to mixed valence vanadium oxides in which both V^5+^ and V^4+^ and even V^3+^ ions are observed. Therefore a large variety of crystalline vanadium oxides have been described. They are usually synthesized via the hydrothermal heating of aqueous solutions [15]. Nanostructured vanadium oxides exhibit unusual morphologies such as nanowires, nanobelts, nanorods, nanotubes and even flower-like or nano-urchin shapes [4,5,6,7,8,9,10,11]. This paper discusses the chemical parameters that are responsible for the formation of a vanadium oxide network from V(V) precursors in aqueous solutions. The molecular structure of these precursors mainly depends on pH but the way they self-assemble depends on the nature of other species in the solution. Supramolecular associations are formed leading to a large variety of crystalline phases. The structure and properties of these nanostructured vanadium oxides have been widely described but the chemical mechanisms leading to their formation from aqueous solutions remain largely unknown. In this paper we would like to draw some relationships between the structure of molecular precursors in aqueous solutions and the nanostructure of vanadium oxides obtained after hydrothermal treatment. This would show how the aqueous chemistry of V(V) can be controlled in order to make nanostructured vanadium oxides, providing an overview of most recent results obtained during the past decade.

## 2. Vanadium (V) Species in Aqueous Solutions

**Figure 1:**
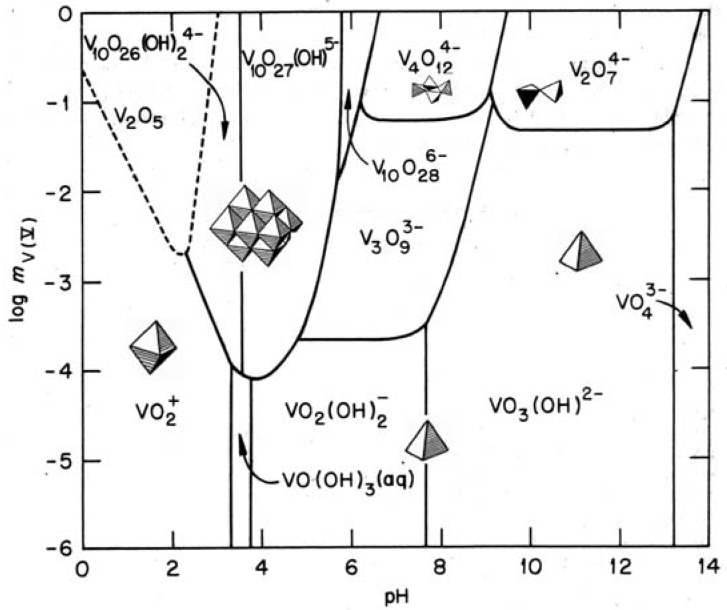
V(V) solute species in aqueous solutions as a function of pH and concentration.

The aqueous chemistry of V(V) has been extensively studied and a large variety of molecular species have been described [16]. At room temperature, they mainly depend on vanadium concentration and pH (Figure 1). Two basic reactions, hydrolysis and condensation, occur when vanadium salt is dissolved in water.

### 2.1. Hydrolysis

In aqueous solutions, V^5+^ ions are solvated by dipolar water molecules giving hydrated [V(OH_2_)_n_]^5+^ species. However, because of the strong polarizing power of the small and highly charged V^5+^ ion, coordinated water molecules are partially deprotonated and the solution becomes more acidic. This hydrolysis reaction can be described as follows:

[V(OH_2_)_6_]^5+^ + hH_2_O ⇒ [V(OH)_h_(OH_2_)_6-h_]^(5-h)+^ + hH_3_O^+^

The hydrolysis ratio ‘h’ increases with pH leading to the formation of aquo, hydroxo and oxo species. It can be easily predicted in the frame of the so-called ‘partial charge model’ based on the electronegativity equalization principle of R.T. Sanderson [17,18]. According to this model, we may assume that an equilibrium is reached when the mean electronegativity χ_p_ of hydrolyzed vanadium precursors [V(OH)_h_(OH_2_)_6-h_]^(5-h)+^ becomes equal to the mean electronegativity χ_aq_ of the aqueous solution. In such solutions, protons are delocalized over the whole network of hydrogen bonds. As a result the electronegativity of solvated protons becomes equal to that of bulk water molecules and the electronegativity of aqueous solutions changes with pH. It decreases linearly with pH as follows:

χ_aq_ = χ_aq_^0^ – λpH

This leads to the following expression [18]:

χ_aq_ = 2.732 – 0.035pH

**Figure 2:**
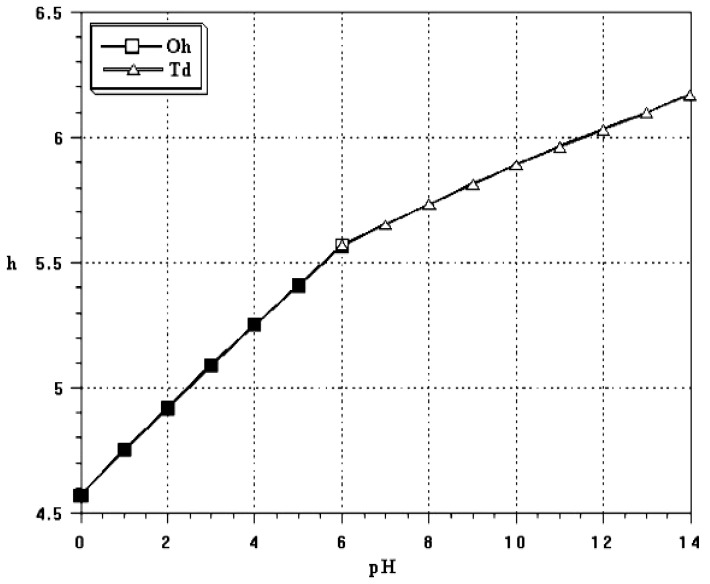
Hydrolysis ratio 'h' of V(V) precursors [V(OH)_h_(OH_2_)_6-h_]^(5-h)+^ as a function of pH.

The mean electronegativity of an aqueous solution decreases linearly with pH from 2.732 at pH 0 down to 2.242 at pH 14 while the hydrolysis ration increases from h = 4.5 [VO_2_(OH_2_)_4_]^+^ to h = 6.2 [VO_3_(OH)]^2-^ (Figure 2).

At low pH (pH < 2), the Partial Charge Model gives an hydrolysis ratio h = 4 and the V^V^ precursor should correspond to [V(OH)_4_(OH_2_)_2_]^+^. However, some internal proton transfer occurs between V-OH groups in order to decrease the positive charge of vanadium. This leads to the formation of vanadyl cations [VO_2_(OH_2_)_4_]^+^ or [VO_2_]^+^ in which two V = O double bonds are formed in cis positions [20].

The coordination number of V(V) decreases from 6 to 4 above pH ≈ 6. Actually the ‘σ’ electron transfer from the coordinated water molecules toward the empty d orbitals of V^5+^ ions increases with deprotonation and the positive partial charge of vanadium decreases. V-O bonds become more covalent and vanadium coordination decreases leading to four-fold coordinated vanadate species [H_n_VO_4_]^(3-n)-^. This coordination change can be easily seen with the naked eyes. The color of V^5+^ (3d^0^) ions is due to electron transfers from the oxygen bonding orbitals to the empty vanadium ‘d’ orbitals. These charge transfer bands move toward UV when the crystal field splitting of ‘d’ orbitals decreases. Six-fold coordinated V(V) decavanadate solutions are typically orange whereas four-fold coordinated metavanadates are colorless [19].

Tetrahedral species [H_n_VO_4_]^(3-n)-^ are then formed. They are progressively deprotonated as pH increases leading to [VO_4_]^3-^ species above pH 12.

### 2.2. Condensation

Actually, monomeric species are only observed in very dilute solutions. Condensation occurs at higher vanadium concentration (Figure 1). Two main reactions are involved in this condensation process.

Olation→-V-OH + -V-OH_2_ ⇒ -V-OH-V- + H_2_O

Oxolation→-V-OH + HO-V- ⇒ -V-O-V- + H_2_O

They both involve the nucleophilic addition of negatively charged OH^δ−^ groups onto positive vanadium cations V^δ+^. Hydroxo V-OH groups are then required for condensation to occur, but olation reactions, in which labile water molecules are already formed, are kinetically faster than oxolation.

At low pH, vanadyl precursors [VO_2_]^+^ cannot lead to condensed species and anions have to be added for precipitation to occur. Vanadyl phosphates VOHPO_4_.nH_2_O, for instance, can be obtained in which [VO_2_]^+^ cations are surrounded by phosphate anions, V-O-V bonds are not formed [21].

**Figure 3:**
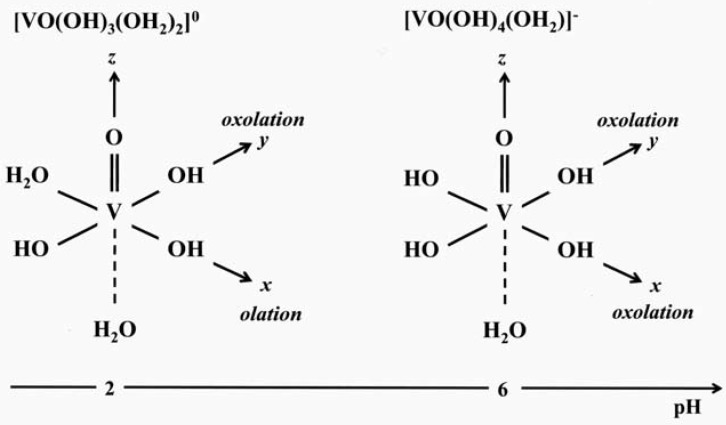
Molecular structure of V^V^ precursors in the pH range 2-8.

The precipitation of vanadium pentoxide V_2_O_5_ occurs at the Point of Zero Charge (PZC), around pH ≈ 2. It should arise from the polycondensation of the neutral precursor [VO(OH)_3_(OH_2_)_2_]^0^. According to its molecular structure, condensation is not possible along the *z* direction O=V-OH_2_. It occurs only within the *xy* plane where V-OH bonds are present (Figure 3). Fast olation reactions along the *x* axis, H_2_O-V-OH lead to chains of edge sharing [VO_5_] square pyramids. These chains are then linked via oxolation reactions along the *y* axis HO-V-OH, leading to vanadium oxide layers made of corner sharing double chains as in orthorhombic V_2_O_5_. This two step mechanism may explain the ribbon-like structure of vanadium oxide gels V_2_O_5_.nH_2_O that have been described as V_2_O_5_ bi-layers made of square pyramidal [VO_5_] units with intercalated water molecules [22,23,24,25].

Negatively charged decavanadate clusters [H_n_V_10_O_28_]^(6^^-n)-^, made of 10 edge-sharing [VO_6_] octahedra, are formed above pH 2. Small and highly charged V^5+^ ions polarize terminal O^2-^ ligands resulting in closed clusters in which M=O bonds point radially toward the outside. Decavanadates behave as strong acids and further condensation does not occur under ambiant conditions. Polyvanadate solid phases are precipitated in the presence of cations. They are built of decavanadate anionic clusters separated by cations [26].

Vanadium coordination decreases above pH ≈ 6 giving tetrahedral anionic precursors [H_n_VO_4_]^(3-n)-^. In the pH range 6–9, difunctional precursors [H_2_VO_4_]^-^ lead to condensed metavanadates forming cycles or chains. Cyclic species such as [V_4_O_12_]^4^^−^ are usually observed in the solution where they can be evidenced by ^51^V and ^17^O NMR [27], whereas chain metavanadates are currently formed in the solid state. They are built up of single chains of corner-sharing [VO_4_] tetrahedra as in KVO_3_ or double chains of edge-sharing [VO_5_] trigonal bipyramids as in KVO_3_·H_2_O. Their formation from cyclic [H_n_V_4_O_12_]^(^^3^^−n)-^ solute precursors can be described via a ring opening polymerization mechanism. The metavanadate salt of ter-butylammonium is especially interesting. Depending on temperature, both cyclic [(CH_3_)_3_CNH_3_]_4_[V_4_O_12_] and chain [(CH_3_)_3_CNH_3_][VO_3_] metavanadates can be synthesized from aqueous solutions. This is the first example of a solid polyoxovanadate precipitated from an aqueous solution containing discrete non protonated [V_4_O_12_]^4^^−^ cyclic anions. A phase transition from cycles to chains can even be observed in the solid state [28].

Further deprotonation leads to [HVO_4_]^2-^ precursors above pH 9. The condensation of these monofunctional species is then limited to dimeric pyrovanadates [V_2_O_7_]^4^^−^ made of two corner-sharing tetrahedra. Fully deprotonated oxo-anions [VO_4_]^3-^ in which V^V^ is surrounded by four equivalent oxygen atoms are observed at very high pH (h = 14). There is no functional V–OH group and V–O–V bonds cannot be formed. Only orthovanadates made of isolated [VO_4_] tetrahedra can be obtained above pH ≈ 12.

### 2.3. Evolution of molecular precursors upon heating

**Figure 4:**
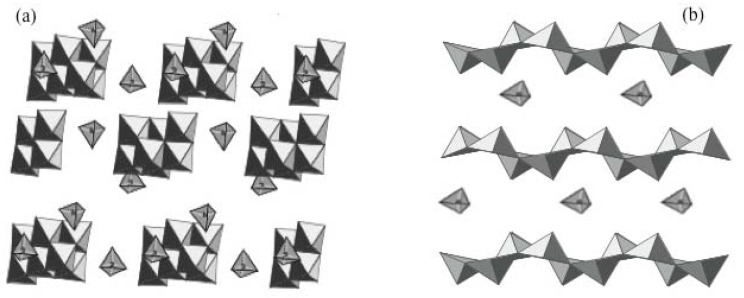
Structure of (a) (TMA)_4_[H_2_V_10_O_28_] synthesized at room temperature and (b) TMA[V_4_O_10_] obtained under hydrothermal conditions.

At room temperature there is a straight correlation between the molecular structure of vanadate precursors in the solution and vanadate anions in the solid precipitate. This is no longer the case when syntheses are performed under hydrothermal conditions. Adding tetramethyl ammonium N(CH_3_)_4_OH (TMAOH) to an aqueous solution of decavanadic acid for instance leads to the precipitation of (TMA)_4_[H_2_V_10_O_28_] made of anionic decavanadate clusters and (TMA)^+^ cations while a layered compound TMA[V_4_O_10_] is obtained, from the same solution, after hydrothermal heating at 180 °C (Figure 4). In both cases, the pH of the solution is the same [15].

**Figure 5:**
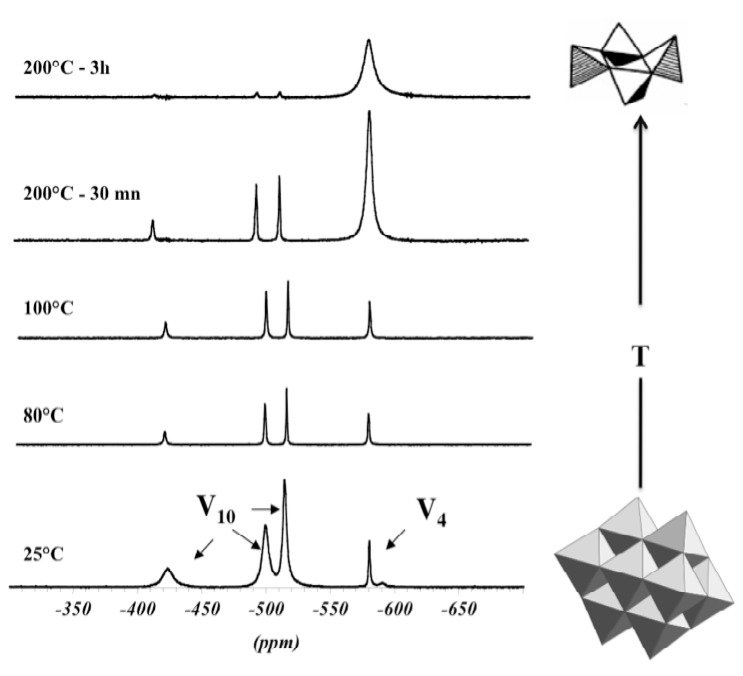
^51^V NMR spectra of a vanadate solution recorded at different temperatures: V_10_ = [H_2_V_10_O_28_]^4-^, V_4_ = [V_4_O_12_]^4-^.

These experiments suggest that the molecular structure of V(V) precursors in aqueous solutions does not depend only on pH, but also on temperature. ^51^V NMR experiments, performed at different temperatures, show that decavanadate solutions progressively transform into cyclic metavanadates [V_4_O_12_]^4-^ upon heating [29]. Only metavanadates are observed around 200 °C (Figure 5). Deprotonation occurs upon heating, leading to the dissociation of decavanadates into metavanadates as follows:

2[H_2_V_10_O_28_]^6-^ + 4H_2_O ⇒ 5[V_4_O_12_]^4-^ + 12H^+^

This reaction is reversible and decavanadates are again observed when decreasing temperature.

In dilute solutions, where only monomolecular species are formed, we may then assume that the deprotonation of the neutral precursor [VO(OH)_3_(OH_2_)_5_]^0^ leads to anionic species such as [VO(OH)_4_(H_2_O)]^-^ that upon dehydration would give more or less protonated tetrahedral vanadate anions [H_n_VO_4_]^(3-n)-^.

[VO(OH)_3_(OH_2_)_2_]^0^ ⇔ [VO(OH)_4_(H_2_O)]^-^ ⇔ [H_n_VO_4_]^(3-n)-^

We could then suggest that upon hydrothermal treatment, solid phases built of [VO_5_] polyhedra are formed via the polycondensation of the intermediate molecular precursor [VO(OH)_4_(H_2_O)]^-^. Its molecular structure shows that oxolation reactions only occur in the '*xy*' plane, along the four V-OH bonds leading to 2D layered compounds rather than ribbon-like particles [Figure 4].

Reduction should also play a role during the thermal transformation of tetramethyl ammonium decavanadates into the layered TMA[V_4_O_10_] oxide [29,30]. Such a transformation is not observed with the inorganic sodium decavanadate. Heating sodium decavanadate directly leads to the stable oxide phases NaVO_3_ and NaV_3_O_8_. The transformation of (TMA)_4_[H_2_V_10_O_28_].4H_2_O into TMA[V_4_O_10_] seems to occur only in the presence of organic cations. ESR experiments show that the transformation does not occur until some V^5+^ are reduced into V^4+^. This reduction, due to the partial decomposition of organic species, favors condensation and coordination expansion, V^4+^ ions being larger than V^5+^ do not adopt tetrahedral coordination.

## 3. Mixed Valence Polyoxovanadate Molecular Clusters

In the previous examples, the formation of polyoxovanadates is governed by the condensation of inorganic precursors. Cations just behave as counter ions in order to get a neutral solid network. Surprisingly foreign anionic species may also behave as templates during the formation of polyoxovanadates. The hydrothermal treatment of V_2_O_5_ and TMAOH leads to the layered compound TMA[V_4_O_10_] but, in the same experimental conditions (temperature and pH), 3D crystals of (TMA)_6_[V_15_O_36_Cl].4H_2_O or (TMA)_10_[H_3_V_18_O_42_I].3H_2_O are obtained in the presence of X^-^ anions (X = Cl, I) [19]. They contain anionic clusters [V_15_O_36_Cl]^6-^ and [H_3_V_18_O4_2_I]^10-^ made of [VO_5_] pyramids linked together to build a hollow cage. The foreign anion is trapped inside this anionic cage whose size depends on the size of the anionic species as shown by the following examples : [CH_3_CN(V_12_O_32_]^4-^, [V_15_O_36_Cl]^6-^, [V_16_O_38_Cl]^8-^, [H_3_V_18_O_42_I]^10-^ and [HV_22_O_54_(ClO_4_)]^6-^ [31,32,33].

The shape of the polyoxovanadate cages also depends on the shape of the anion. (ClO_4_)^-^ leads to the formation of spherical cages such as [HV_22_O_54_(ClO_4_)]^6-^ while elongated azide anions (N_3_)^-^ lead to ellipsoidal [H_2_V_18_O_44_(N_3_)]^5-^ cluster cages [34]. The mixed valence polyoxovanadate [V_34_O_82_]^10-^ exhibits quite a strange molecular structure. It is made of an ellipsoid-shaped [V_30_O_74_] sheath built of 30 tetragonal [VO_5_] pyramids surrounding a central [(V_4_O_4_)O_4_] cube [35].

These polyoxvanadate cages can be viewed as sections of layers of vanadium pentoxide V_2_O_5_. The condensation of V–OH groups in the equatorial plane of the [VO(OH)_4_(OH_2_)]^-^ precursor leads to the formation of 2D compounds made of corner and edge-sharing [VO_5_] pyramids. However, the way these pyramids are linked together depends on the nature of the other ionic species in the solution [36].

Large and weakly polarizing tetramethyl cations interact weakly with the negative oxygen of terminal V = O^δ−^. They only behave as counter cations in order to form a neutral solid with the anionic polyoxovanadate network. Interactions between adjacent V = O dipoles lead to a layered structure with [VO_5_] pyramids alternatively ‘‘up’’ and ‘‘down’’.

**Figure 6:**
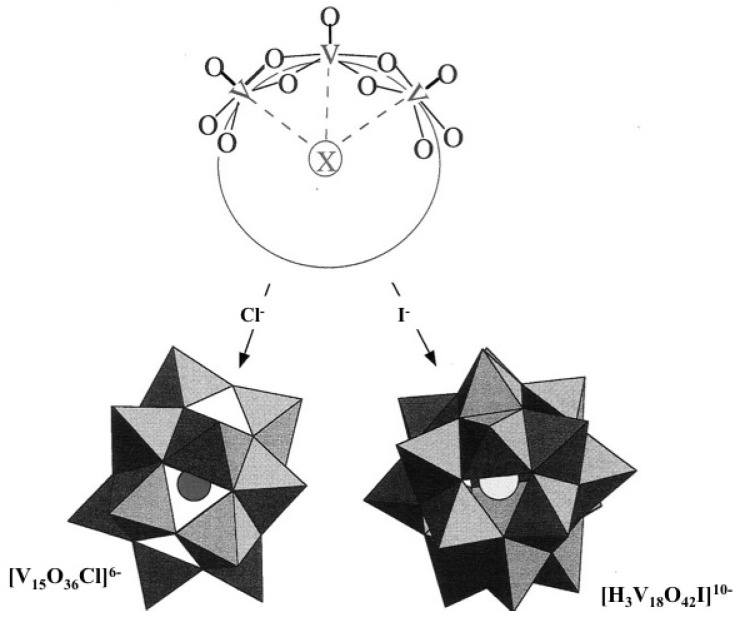
Polyoxovanadate clusters formed in the presence of X^-^ anions [V_15_O_36_Cl]^6-^ and [H_3_V_18_O_42_I]^10-^.

This is no longer the case with smaller and more polarizing inorganic anions (Cl^-^, I^-^, NO_3_^-^,...). The specific role of these anions should be due to the particular geometry of [VO_5_] pyramids in which the weakly bonded water molecule V-OH_2_ opposite to the V = O bond is highly labile. Negative anions X^-^ can then interact with the positive V^5□^ along the *z* axis, leading to the curved condensation of [VO_5_] square pyramids around this central anion to form a closed cage [37,38]. All V = O dipoles are then oriented toward the outside of the shell where they repeal each other, leading to the formation of convex surfaces (Figure 6). Interactions between the central anion and the negatively charged polyoxovanadate cage are very weak. The vanadate cage is mainly polarized by the central anion so that the negative density is pushed toward the V = O pointing outside of the cage where they interact with the positive cations [39].

## 4. Nanostructured Vanadium Oxides

### 4.1. From 1D to 2D oxides

A large number of nanostructured vanadium oxides have been described during the past few years. They are synthesized via the hydrothermal treatment of aqueous solutions of V(V) precursors. Their morphology is usually related to the layered structure of orthorhombic V_2_O_5_. Therefore 1D and 2D structures such as nanowires, nanofibres, nanorods, nanoribbons, nanobelts or nanosheets are typically reported in the literature. Temperature and pH appear to be the main parameters to control the morphology of these V_2_O_5_ based nanomaterials [19].

Heating V_2_O_5_ with TMAOH for instance leads to different layered compounds depending on pH [14]. Ribbon-like (TMA)[V_8_O_20_] particles are obtained at pH 3. They exhibit a 2D structure made of chains of edge-sharing [VO_6_] octahedra joined together by corners to form [V_8_O_20_] sheets. Plate-like (TMA)[V_4_O_10_] particles are obtained at pH 6. Oxide layers are made up of double chains of edge and corner sharing [VO_5_] square pyramids linked together by corners. In both cases, the structure is close to that of orthorhombic V_2_O_5_ and the main difference between both polyvanadates is the shape of the particles. Their width increases with pH, platelets are formed at pH 6 and ribbons at pH 3. This could be explained by looking at the structure of the hydrolyzed molecular precursors (Figure 3). Around pH 3, the main molecular precursor (h = 5) should be [VO(OH)_3_(OH_2_)_2_]^0^. Olation reactions along V-OH_2_ bonds are faster than oxolation reactions along V-OH bonds, leading to the anisotropic growth of (TMA)[V_8_O_20_] nanobelts or nanoribbons. The neutral precursor undergoes deprotonation when pH increases giving the anionic molecular species [VO(OH)_4_(OH_2_)]^-^ around pH 6. Only oxolation reactions then occur in the *xy* plane along the four equivalent V-OH bonds leading to plate-like (TMA)[V_4_O_10_] particles that exhibit a truly 2D structure [40]. Above pH≈6, V^5+^ ions become tetrahedrally coordinated and the resulting polyoxovanadate (TMA)[V_3_O_7_] obtained at pH 8 is a truly 2D oxide with V_3_O_7 _layers made of zig-zag chains of edge-sharing [V^4+^O_5_] square pyramids connected by corner-sharing [V^5+^O_4_] tetrahedra [41].

In all cases, large alkylammonium cations are just intercalated between the oxide layers. Some reduction occurs during the thermal treatment leading to mixed valence compounds that contain both V^5+^ and V^4+^ ions. The number of reduced vanadium ions increases with pH. The ratio V^4+^/V^5+^ = 1/7 at pH 3, 1/3 at pH 6 and 2 at pH 8 [15].

A large variety of polyoxovanadates exhibiting 1D or 2D structures have been reported during the past decade. As a general rule, they progressively turn from 1D to 2D as pH increases. As shown just before, this should result from the deprotonation of the fourth water molecule in the equatorial *xy* plane. Condensation via olation progressively decreases. Depending on the experimental conditions (pH and temperature) this leads to the formation of nanowires [42,43,44,45], nanofibres [46,47], nanoribbons [48], nanorods [49,50,51,52,53], nanobelts [54,55,56,57,58,59,60,61,62] and nanosheets [63].

The case of H_2_V_3_O_8_ nanobelts is especially interesting. These nanobelts are obtained via the hydrothermal treatment of V_2_O_5_ suspensions (190 °C, 24 h). A pure orthorhombic crystalline phase (JCPDS 89-0612) is obtained. According to authors, the width of H_2_V_3_O_8 _nanobelts can be controlled by adjusting the pH value. It_. _increases from 100 nm at pH 3 up to 1 µm at pH 5 [64,65,66,67].

### 4.2. Vanadium oxide nanotubes

Vanadium oxide VO_x_ nanotubes were discovered by R. Nesper and co-workers [5,6]. They are several µm in length and offer a typical example of multiwalls tubular structures. Their walls may contain up to 30 vanadium oxide layers, giving an outer diameter up to 100 nm. Many very good papers have been published describing their structure [68,69,70] and physical properties: electronic [71,72,73,74], optical [75,76], or magnetic [77]. However the main interest for these nanotubes remains their electrochemical properties. The template organic molecules can be removed without breaking the structure of the nanotubes that remain redox-active allowing the reversible insertion of Li^+^ ions [78,79,80,81,82,83,84].

**Figure 7:**
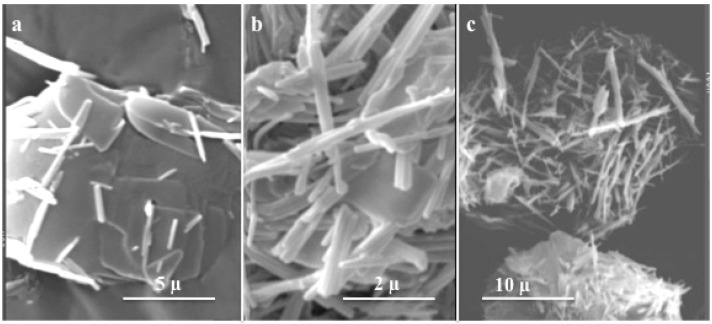
Formation of VOx nanotubes upon hydrothermal treatment of V_2_O_5_ gels at 180C: (a) after 8h (b) after 66h ( c) after 120 h.

VOx nanotubes are currently formed via hydrothermal syntheses (≈180 °C, few days) in the presence of long chain alkyl amines such as HDA (HexaDecyl Amine). Different precursors have been used such as vanadium pentoxide V_2_O_5_ [85,86], vanadium alkoxides VO(OR)_3_ [68,79], vanadium oxychloride VOCl_3_ [87], ammonium metavanadate NH_4_VO_3_ [88] or vanadium oxide gels V_2_O_5_,nH_2_O [89,90]. In all cases, layered vanadium oxides are formed in the aqueous solution that then roll up into nanotubes [91,92]. A mixture of vanadium oxide nanotubes and unrolled vanadium oxide sheets can be observed during the hydothermal treatment, confirming the link between nanotubes and exfoliated lamellar intermediates [93]. The number of plate-like particles progressively decreases with time while more nanotubes are formed and only nanotubes are observed after few days (Figure 7).

Actually two main chemical processes seem to be involved in the formation of VOx nanotubes, namely the intercalation of organic molecules between the oxide layers and the reduction of V^5+^ ions.

Vanadium oxide is known to be a typical layered material. A wide range of molecular species can be intercalated between the oxide layers. The basal distance increases upon intercalation decreasing the interactions between oxide layers. A swelling process is even observed with vanadium pentoxide xerogels leading to the formation of colloidal solutions made of exfoliated V_2_O_5_ layers dispersed in water [22,93]. These oxide layers can then behave almost freely and roll up into curved structures such as nanorods, nanotubes or nanoscrolls [92,94,95,96,97,98].

Intercalation appears to be an important step during the formation of nanotubes. Before hydrothermal treatment, V_2_O_5_ and alkylamine mixtures have to be aged at room temperature in order to induce the intercalation process [5,68]. The size of intercalated organic molecules seems also to be important to decrease interaction between oxide layers. VOx nanotubes are obtained in the presence of long chain alkyl amines while smaller amines do not lead to the same morphology [99]. Vanadium oxide nanotubes about 120 nm in diameter are observed in the V_2_O_5_-HDA system while only nanorods (diameter ≈ 20 nm) are formed with V_2_O_5_-EtOH [100]. It has to be pointed out that these intercalated amines can be exchanged with metal cations in the solution [101].

Organic molecules also favor the reduction of the oxide as shown by the color of the sample that turns from orange to green and black. This leads to the formation of a mixed valence compound that contains both V^5+^ and V^4+^ ions (about 50%) [102]. Actually, VOx nanotubes can also be obtained via the oxidation od V^4+^ solutions [103]. Reduced V^4+^ ions are much bigger than V^5+^ (r_V4+_ = 0.85 Å, r_V5+_ = 0.49 Å). This can cause a significant degree of stress favoring the curvature of the oxide layers. Moreover, temperature favors the formation of tetrahedral [VO_4_] vanadium species. It has been shown that the structure of these nanotubes is close to that of BaV_7_O_16_. It is made of two sheets of [VO_5_] square pyramids pointing in opposite directions and connected by [V^5+^O_4_] tetrahedra [104]. These tetrahedra prevent electron delocalization all over the sample. A strong electron-phonon coupling is observed leading to the local distortion of V^4+^ sites with the formation of small polarons [105].

### 4.3. Nanostructured oxides

The hydrothermal synthesis of nanostructured vanadium oxides currently leads to 1D material such as nanotubes, nanorods, nanowires or nanobelts. However one further step could be obtained via the self assembling of these 1D structures into curved structures such as spheres, flower-like or nano urchins.

V_2_O_5_ nanorods for instance lead to the formation of ellipse-like macro-plates in which they lie parallel or perpendicular to each other [106]. Vanadium oxide bundles have been obtained via the sonication of V_2_O_5_-H_2_O suspensions. These spindle-like V_2_O_5_ bundles are made of several tens of 1D nanoparticles (nanowires or nanorods), with diameters 30-50 nm and lengths of 3-7 µm. Ultrasounds induce the dispersion of the oxide layers than can roll up into wires or rods and then self-assemble side-by-side into bundles [107,108]. Foreign ions such as Co^2+^ ions have been shown to be able to behave as nucleation centers. They lead to the formation of bundle-like V_2_O_5_.xH_2_O nanostructures made from nanobelts synthesized via a simple hydrothermal treatment of V_2_O_5_ and H_2_O_2_ [109].

Hollow V_2_O_5 _microspheres have been obtained via the self-assembly of nanorods [110]. These nanorods about 200 nm in diameter and up to 2 µm long are formed when vanadium acetylacetonate [V(acac)_3_] is heated with ethylene glycol. They self-assemble into hollow microspheres in the presence of poly(vinylpyrrolidone) (PVP). Ethylene glycol has been shown to give metal oxide nanowires [111,112]. Therefore the formation of rod-like vanadium precursors could be explained via the coordination of EG giving a vanadium glycolate followed by an oligomerization reaction. PVP then behaves as a template in which vinyl groups are hydrophobic while carbonyl groups are hydrophylic, leading to the formation of micelles. Vanadium oxide microspheres, made of closely packed particles and radially aligned rods, are also formed in the presence of oxalic acid. [113,114,115]

Roselike nanostructured vanadium oxide films have been synthesized from water-ethanol solutions of vanadium tri-isopropoxide VO(OPr^i^)_3_, using hexadecylamine as a template. These films are obtained by drop-casting of the solution onto Si wafers. They are made of radially packed petal-like flat structures giving rise to spherical aggregates about 40 nm in diameter. These films exhibit photo‑induced hydrophilic properties. They can switch reversibly between superhydrophobic and superhydrophilic under UV irradiation and dark storage respectively [116]. Similar roselike nanostructures have also been obtained via the thermal decomposition of ammonium vanadium sulfate hydroxide [NH_4_V_3_(OH)_6_(SO_4_)_2_]. This compound synthesized via the hydrothermal treatment of NH_4_VO_3 _with oxalic acid in a DMSO-H_2_O solvent is made of flat particles that self-assemble into a rose-like structure. A pure V_2_O_5_ phase is obtained upon calcination at 500 °C without destroying the flower morphology [117]. Such flower-like nanostructures are not limited to V(V) oxides, nanostructured reduced vanadium oxides such as VO_2_ petaloid clusters or VOOH hollow dandelions have also been described recently [118,119].

The most amazing nanostructured vanadium oxide described during the past few years should be the spherical clusters that look like 'nano-urchins' [120,121]. They have been obtained via the hydrothermal treatment of an ethanolic solution of vanadium tri-isopropoxide and alkyl amines. A layered compound is first formed with alkyl amines intercalated between the oxide layers. These oxide lamina self-organize into a fan-like laminar structure, presumably because of the presence of non intercalated free amines. They then roll up to form hollow nanotubes that can be up to several micrometers in length. The tube walls are made of vanadium oxide layers with intercalated organic surfactant molecules. The radial self-organization of VO_x_ nanotubes leads to the formation of spherical structures that look like nano-urchins. Almost perfect scrolled layers are formed under highly reducing conditions when the amine intercalation is maximized while 'nano-urchin' structures are formed under less reducing conditions [122,123]. Amazing six-fold rotationally symmetric vanadium oxide nanostructures have been obtained from V_2_O_5_ xerogels treated with dodecanethiol. Each nanostructure is made of six spoke-like V_6_O_11_ platelets making an angle of about 60°. Their formation from the layered xerogel is explained via a morphotropic transition [124].

**Figure 8:**
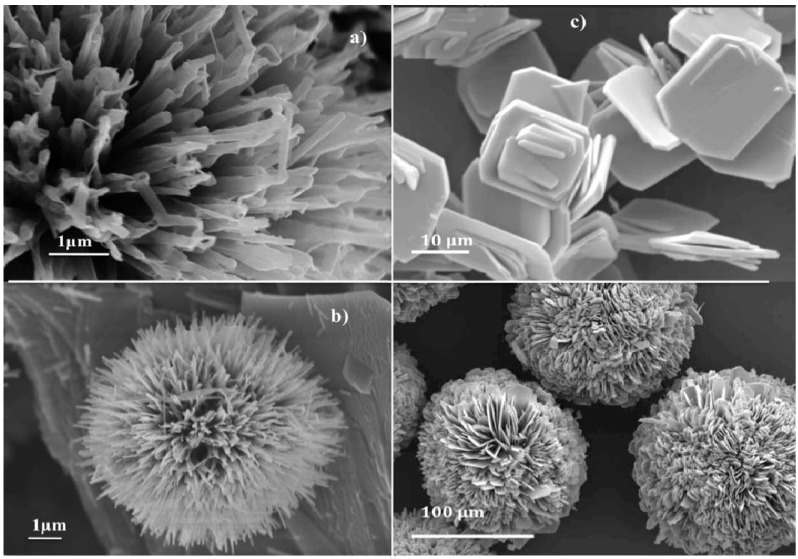
Nanostructured vanadium oxides obtained via the self-assembling of nanoparticles: a) and b) Nano-urchin from VOx nanotubes, c) and d) nanospheres from platelets hexavanadate nanoparticles Cs_2_V_6_O_16_.

## 5. Conclusion

Nanostructured vanadium oxides have been widely studied during the past decade. Many syntheses, such as chemical vapor deposition [125] or anodic deposition, also lead to amazing morphologies [126,127]. However, most syntheses are based on the hydrothermal treatment of aqueous solutions. These nanostructured vanadium oxide based materials range from molecular polyoxovanadate clusters to nanotubes and even nano-urchins. Some strong correlations can be drawn between the molecular structure of solute precursors in the solution and the nanostructure of the resulting materials. Their analysis should lead to a better control of the synthesis of these nanostructured vanadium oxides. Temperature, pH, foreign organic or inorganic species are some of the main parameters. The structure of nano-urchins, nanotubes and nanorods for instance depends strongly on the valency of vanadium and its interactions with the organic surfactants [122,123].

Nanostructured vanadium oxide exhibits specific properties that could be used for the realisation of new devices. The role of nanostructure in improving the performance of electrodes for energy storage and conversion has been clearly demonstrated [128,129]. The electrochemical insertion of Li^+^ ions for instance appears to be much easier and reversible into nanostructured vanadium oxides than in bulk V_2_O_5_, a property that could be exploited for lithium batteries. Open-ended multiwalled nanotubes have been shown to exhibit improved electrochemical properties. They have a high initial discharge capacity of 457 mAh^-1^ and good cycling performances [79,83].

Well ordered VOx nanorolls are obtained under highly reducing conditions. Their electrochemical responses are similar to those of crystalline V_2_O_5_. A significant increase in specific capacity is observed with defect-rich vanadium oxide nanorolls obtained under less reducing conditions. The association of nanoscale and atomic scale disorder appears to produce the best combination of increased specific capacity and better cyclability. Therefore, the unprecedented density of urchin’s nanotubes, could open new possibilities for making lithium nano-batteries [130].
